# Valorization of Olive Pomace-Based Nutraceuticals as Antioxidants in Chemical, Food, and Biological Models

**DOI:** 10.3390/molecules23082070

**Published:** 2018-08-18

**Authors:** Dubravka Vitali Čepo, Kristina Radić, Sanja Jurmanović, Mario Jug, Marija Grdić Rajković, Sandra Pedisić, Tihomir Moslavac, Petra Albahari

**Affiliations:** 1 Faculty of Pharmacy and Biochemistry, University of Zagreb, Ante Kovačića 1, 10000 Zagreb, Croatia; kradic@pharma.hr (K.R.); sjurmanovic@gmail.com (S.J.); mjug@pharma.hr (M.J.); mgrdic@pharma.hr (M.G.R.); saler_petra@yahoo.com (P.A.); 2 Department of Food Engineering, Faculty of Food Technology and Biotechnology, University of Zagreb, Pijerottijeva 6, 10000, Zagreb, Croatia; spedisic@pbf.hr; 3 Faculty of Food Technology, University in Osijek, Franje Kuhača 20, 31000 Osijek, Croatia; tihomir.moslavac@ptfos.hr

**Keywords:** olive pomace, cyclodextrin encapsulation, antioxidant activity, meat model, β-carotene linoleate model, inhibition of DNA scission, liposome model

## Abstract

Waste remaining after the production of olive oil (olive pomace) is known to contain significant amounts of phenolic compounds that exert different types of biological activities, primarily acting as antioxidants. In this work, a sustainable approach that combines ultrasound-assisted extraction with food-grade solvents and encapsulation with different types of cyclodextrins was used to prepare olive pomace-based polyphenol rich extracts that were tested as antioxidants in various chemical, food, and biological model systems. Encapsulation with cyclodextrins had a significant positive impact on the chemical composition of obtained extracts and it positively affected their antioxidant activity. Observed effects can be explained by an increased content of polyphenols in the formulations, specific physical properties of encapsulated compounds improving their antioxidant activity in complex food/physiological environment, and enhanced interaction with natural substrates. Depending on the applied model, the tested samples showed significant antioxidant protection in the concentration range 0.1–3%. Among the investigated cyclodextrins, hydroxypropyl-β-cyclodextrin and randomly methylated-β-cyclodextrin encapsulated extracts showed particularly good antioxidant activity and were especially potent in oil-in-water emulsion systems (1242 mg/g and 1422 mg/g of Trolox equivalents, respectively), showing significantly higher antioxidant activity than Trolox (reference antioxidant). In other models, they provided antioxidant protection comparable to commonly used synthetic antioxidants at concentration levels of 2–3%.

## 1. Introduction

The production of the olive oil leads to large amounts of waste known as olive pomace and olive mill wastewater. Since it needs to be adequately processed before disposal to the environment, it creates considerable economic and ecological burden [1]. Therefore, a suitable use of these residues could positively affect economic status of the olive oil producers and reduce the negative impact on the environment. However, only small amounts of olive mill waste are re-used, mainly as fertilizers, biomass, or additive in animal feed, while a large quantity remains without actual application [2].

Olive pomace is a heterogeneous mixture of many chemical compounds, such as metals, sugars, and polyphenols [3]. Polyphenols are particularly abundant, since, only 2% of them is transferred to the olive oil during production and the rest retained in the pomace, making it an interesting alternative source of phenolic compounds [4].

Phenolic compounds are the main antioxidant compounds in olive pomace. When consumed, a large amount of them is hydrolyzed into hydroxytyrosol, which is then absorbed in the small intestine. Hydroxytyrosol is a powerful antioxidant, exerting a wide variety of biological effects. So, hydroxytyrosol derivatives are considered the main bearers of pharmacological effects of extra virgin olive oil [5]. It is well known that dietary antioxidants protect the body from free radicals which are constantly formed during physiological processes. Imbalance between the production of free radicals and the antioxidant defense system is responsible for the occurrence of oxidative stress which is related to different physiological and pathological processes such as ageing, cancer, cardiovascular diseases, and diabetes [6,7]. A large number of epidemiological studies indicates the existence of an inverse relationship between dietary intake of foods rich in antioxidants and the incidence of the abovementioned diseases [8]. New research also shows that olive-derived polyphenols can exert particular pharmacological effects by other mechanisms, such as participating in the activation of different signaling pathways involved in the prevention of inflammation, oxidative stress, or insulin resistance [9]. Polyphenols can also be used by the food industry as natural food additives with antioxidant properties and be used for extending the shelf life of food and reducing nutritional losses and formation of harmful substances. This is especially important nowadays when increased nutritional intake of certain synthetic antioxidants such as butylated hydroxyanisole (BHA) and butylated hydroxytoluene (BHT) is potentially linked to particular safety issues [10,11]. Therefore, currently, the development and utilization of effective but non-toxic antioxidants of natural origin is the area of an extensive research. In this sense, investigation of the possibility of recovering a polyphenol-rich extract from olive pomace—a low-cost and widely available by-product—presents a logical and sustainable approach. An effective search of sources of naturally occurring antioxidants and sustainable design and validation of novel functional formulations require simple and available but reliable methods of antioxidant activity evaluation.

To this day, many chemical-based (in vitro) methods have been developed for measuring the efficiency of different types of antioxidants. They can be divided into two major groups: hydrogen atom transfer reactions (oxygen radical absorbance capacity (ORAC) or carotene bleaching) and electron transfer reactions (Trolox equivalent antioxidant capacity (TEAC) or diphenyl- picryl-hydrazyl radical (DPPH**˙**) scavenging assay) [12]. These methods are inexpensive, useful for screening, show high-throughput, and produce an index value that allows comparison between different products. However, they are performed in the absence of lipids and do not evaluate the partitioning of antioxidants, leading to a lack of correlation with the actual performance of particular antioxidants in food or biological systems.

In order to additionally improve the accuracy of in vitro testing, new models have been developed that simulate the chemical, physical, and environmental conditions similar to foods or biological matrices [13]. Several types of food models can be used: bulk oil, oil-in-water emulsions, and a model of muscle food. Compared to chemical methods, biological models (like liposome membrane system or methods utilizing plasmid DNA) that apply natural substrates and mimic physiological environment could more accurately predict the activity of tested compounds in biological systems. Unlike cell-culture methods, they still only take into account the ability of antioxidant to scavenge free radicals, and cannot assess other aspects of antioxidant activity, such as upregulation of antioxidant and detoxifying enzymes, modulation of redox cell signaling and gene expression, etc. However, in comparison to widely used cell culture methods, they are still significantly faster, simpler, and cheaper and therefore more applicable for screening or during earlier phases of product development. Therefore, they should be used in combination or instead of chemical-based methods in order to provide more accurate information about tested compounds/extracts. Namely, only proper selection of the method or more methods can provide accurate assessment of antioxidant activity and give reliable information about the potential of the antioxidant as a food preservative or health-promoting agent.

An additional problem in the development of novel formulations from complex plant-based extracts is the fact that they frequently occur as a sticky material of poor stability and poor solubility with penetrating smell and unpleasant taste, all presenting practical difficulties for their validation and industrial use [14]. Utilization of an efficient carrier system during the extraction and/or drying process is therefore necessary to ensure adequate powder consistency, and to increase the stability of active compounds during drying and storage. Previously, Albahari et al. [15] showed that application of cyclodextrins during production of olive pomace extracts significantly improved extraction yields, powder properties, and stability during storage. By enhancing the content and stability of active compounds in the formulation and by modulating their release and availability [16], cyclodextrins could significantly affect the functionality of the final product. Increasing the reuse of olive waste by producing novel added-value products might result in both environmental and economic benefits. To do that, simple and sustainable processes of extraction and product formulation need to be developed. Also, possible areas of application (dietary supplement, natural food additive, etc.) need to be defined more specifically so that targeted formulation design can be applied, resulting in optimal product characteristics. Therefore, the aim of this study was to test the applicability of dried olive pomace extracts (DOPE) as antioxidants in different food and biological model systems and to assess the impact of different cyclodextrins on the activity of developed formulation. To our knowledge, until now, olive pomace extracts were tested as antioxidants only in common chemical-based tests and the current application of cyclodextrins in complexation of bioactive compounds present in olive waste is mainly focused on the study of interaction between the selected components with naturally occurring cyclodextrins. Obtained data will provide novel information about the possible applications of processed olive waste and mechanisms of their antioxidant action and elucidate potential benefits of utilizing cyclodextrin-encapsulation as a simple and cost-effective approach for improving the functionality of complex natural extracts.

## 2. Results and Discussion

### 2.1. Chemical Composition and Antioxidant Activity in Chemical Model Systems

#### 2.1.1. Chemical Composition of DOPE

Olive pomace extracts were obtained by applying ultrasound-power and using only food-grade solvents (the mixture of ethanol and water), according to previously optimized procedure [14]. As presented in Table 1, the most abundant polyphenolic compounds in the obtained extract were hydroxytyrosol derivatives, hydroxytyrosol, tyrosol, and oleuropein, with concentrations ranging from 16.29 to 51.29 mg/100 g. The amount of simple phenolic acids (3,4-dihydroxybenzoic acid, vanillic acid, homovanillic acid and *p*-hydroxybenzoic acid) was significantly lower, ranging from 0.33 up to 3.72 mg/100 g.

Obtained results are generally in agreement with data available for the composition of olive pomace published by other authors. Majority of authors point out hydroxytyrosol, tyrosol and oleuropein as major polyphenolic compounds in olive pomace; however, oleuroepin is usually the most represented one [17,18,19]. In our case, the amount of oleuropein was significantly lower in comparison to the amounts of hidroxtyrosol or tyrosol. This is not in accordance with previously obtained data about the content of hydroxytyrosol derivateives in olive pomace extracts produced from the pomace collected from two-phase mills that contained significantly higher content of oleuropein (1090 mg/kg) [15]. Similarly, Cioffi et al. [18] reported significantly lower hydroxytyrosol and tyrosol content of olive pomace obtained from two olive cultivars (8.4–10.4 mg/kg and 20.7–21.6 mg/kg, respectively) in comparison to oleuropein content (81.7–83.0 mg/kg). Aliakbarian et al. [17] analyzed the chemical composition of olive pomace extracts obtained in high-pressure-high temperature reactor and reported relatively low hydroxytyrosol and tyrosol content (21.9 and 21.1 mg/100g dry matter, respectively) and high oleuropein content (203.1 mg/100 g dry matter) in extract obtained under conditions similar to ours.

Observed discrepancies can be explained by the fact that oleuropein content in olives and olive by-products can be significantly influenced by various factors such as olive cultivar, maturation stage, processing conditions etc. [20]. In our case, it is important to state out that pomace has been collected from the mills during February when majority of fruits is in its black maturation phase that is characterized by hydrolysis of components with higher molecular weight (such as oleuropein) and subsequent formation of tyrosol and hydroxytyrosol [21].

Independent of raw material characteristics, the chemical composition of olive pomace extract can be significantly affected by the type of extraction, drying process, and the presence of different carrier systems during product development [15]. Impact of the utilization of different types of cyclodextrins during formulation of DOPE on the polyphenolic content and composition is presented in Table 2.

As shown, the presence of cyclodextrins in the extraction solvent significantly affected the content of hydroxytyrosol, tyrosol, and oleuropein in DOPE, which is consistent with our previous investigations [15]. Randomly-methylated β cyclodextrin (RAMEB) and hydroxypropyl-β-cyclodextrin (HPβCD) significantly increased hydroxytyrosol yields, RAMEB increased tyrosol content, while HPβCD significantly increased oleuropein yields. When taking into account total hydoxytyrosol derivatives (HTD) content, RAMEB and HPβCD can be considered the most efficient among the tested cyclodextrins.

Observed results are consistent with previous findings pointing out β-cyclodextrins with the central cavity diameter of 8 Å, as the most appropriate for the inclusion of antioxidant compounds from the olive oil [22,23]. Tyrosol was also proved to form stabile inclusion complexes with native βCD and its 2-hydroxypropyl- and methylated- derivatives [24]. Complexation of hydroxytyrosol with βCD also provided a strong photo-protection of the polyphenolic compound upon irradiation with UV (λ = 254 nm). Finally, oleuropein (OLE) also forms stabile inclusion complexes with βCD, increasing its aqueous solubility by 50% and protecting it against oxidation, as demonstrated by DSC studies under oxidative conditions [25]. In our study, only the oleuropein content of the obtained DOPE was positively affected by utilization of γCD. This particular effect on oleuropein might be explained by the larger central cavity of γCD (in comparison to βCDs), making it suitable for formation of an inclusion complex with oleuropein but not with smaller (tyrosol and hydroxytyrosol) molecules.

Formation of inclusion complexes increases solubility of target compounds in aqueous solvents (such as water–ethanol mixtures used in this investigation) and protects target compounds during drying process resulting with increased content in the final product.

#### 2.1.2. Antioxidant Activity of DOPE in Chemical Models

The impact of the addition of cyclodextrins on antioxidant activity determined in chemical models (total reducing capacity (TRC) and antiradical efficiency) is presented in Table 2. Total reducing capacity of analyzed samples ranged from 39.34 mg/g GAE in nDOPE up to 53.16 mg/g in RAMEB encapsulated DOPE (ramDOPE). Statistical analysis showed that TRC of all cyclodextrin-encapsulated DOPEs was significantly higher in comparison to the native sample, but especially in HPβCD encapsulated DOPE (hpDOPE) and ramDOPE. Scavenging capacity against free radicals was measured utilizing 2,2′-azino-*bis*-(3-ethylbenzothiazoline-6-sulphonic acid) radical cation (ABTS^+˙^) and DPPH**˙** and by the ORAC method. A significant increase of ABTS^+^ scavenging activity was achieved by hpDOPE and ramDOPE and increase of DPPH**˙** scavenging activity was achieved by hpDOPE, ramDOPE, and γCD encapsulated DOPE (gDOPE). Similar results were obtained by DPPH methods—ramDOPE and hpDOPE had the lowest EC50 values among the tested samples, while encapsulation with βCD or γCD produced less significant or no effect.

Enhanced antiradical activity against ABTS^+˙^ or DPPH**˙** can be explained by increased content of hydroxytyrosol derivatives (HTD) in CD-containing extracts (Table 2). Namely, hydroxytyrosol, tyrosol, and oleuropein have previously been identified as the principal antioxidative compounds in of olives and derived products [1,4]. Additionally, the antioxidant activity of complexed polyphenols can be increased because in the form of the inclusion complex, there are protected against the rapid oxidation by free radicals [26].

ORAC assay was used for the measurement of scavenging activity of DOPE against peroxyl radical scavenging activity. Since it is simple method, adoptable for both hydrophilic and lypophilic antioxidants, reproducible, utilizes a biologically relevant radical source, and is adaptable for routine quality control analyses, it has been considered as a serious candidate as a standardized AOC method for antioxidant capacity of foods and dietary supplements [27]. Also, as opposed to methods that utilize fixed end-point measurement, ORAC takes into account different kinetic behaviours of the antioxidants, providing more complete information about antioxidant behavior [28]. Therefore, despite criticism and recent challenges [29,30], it is still among the most widely used methods for the measurement of antioxidant capacity in foods. The antioxidant activity of the samples was calculated from the net area under the curve (nAUC) obtained by measuring the fluorescence of samples (and blank) for 45 min. A standard curve was generated by plotting the nAUC against corresponding Trolox concentrations.

As can be seen from the presented data (Table 2), ORAC values in analysed samples ranged from 142.7 mg/g TE in bDOPE up to 166.5 mg/g TE in hpDOPE. Although antioxidant activity was higher in hpDOPE and ramDOPE in relation to native DOPE (nDOPE) (which is consistent with the data obtained by other applied methods), the observed changes were not statistically significant. The obtained results indicate that under more realistic conditions where antioxidants are expected to work during a significant amount of time, positive effects of HPβCD or RAMEB could be less significant than indicated by TRC, TEAC, or DPPH radical scavenging activity measurements.

However, the obtained results generally underline the benefits provided using cyclodextrins as extraction enhancers and the spray drying additives. During extraction, cyclodextrins can increase the extractability of certain polyphenolic compounds by forming inclusion complexes that can increase the solubility of a target compound in hydrophilic solvents and increase its stability during the extraction process. Formation of inclusion complexes between olive-derived phenolic compounds, such as oleuropein, tyrosol, and different types of cyclodextrins, has been proven by several authors [24,31]. Also, encapsulated within the cavity of the cyclodextrin molecule, the stability of the target compound during extraction or drying process is additionally protected.

Our results are consistent with the aforementioned observations and point out the particular advantages of HPβCD and RAMEB in relation to other tested cyclodextrins (CDs). This is consistent with our previous investigation [15] and might be explained by the preferential steric compatibility and higher complex stability of major polyphenols in olive pomace with HPβCD and RAMEB over other tested CDs. Therefore, hpDOPE and ramDOPE were tested as antioxidants in different food and biological model systems and their activity was compared to the activity of nDOPE and commonly used synthetic antioxidants (BHA or Trolox).

### 2.2. Antioxidant Activity of DOPE in Food Model Systems

#### 2.2.1. Antioxidant Activity of DOPE in Oil

Oxidation of lipids is the major cause of oil quality reduction, leading to formation of toxic compounds and off-flavours. The oxidative stability of oil depends primarily on its fatty acid composition and the presence of anti- and pro-oxidative concomitant compounds and interactions between them [32]. Oxidation proceeding in oils may be inhibited by the application of various methods decreasing air content in the package, including inert gases: nitrogen and carbon dioxide, and elimination of oxygen, e.g. oil flushing with a specified gas and/or formation of a gas cushion above the oil in the package. Addition of synthetic antioxidants (such as BHT or propylgallate (PG)) to oil is also an efficient and widely accepted approach. However, since the safety of the mentioned synthetic antioxidants has recently been questioned, there is increasing demand to find safer natural alternatives that can be produced by sustainable methods from available resources.

**Figure 1:**
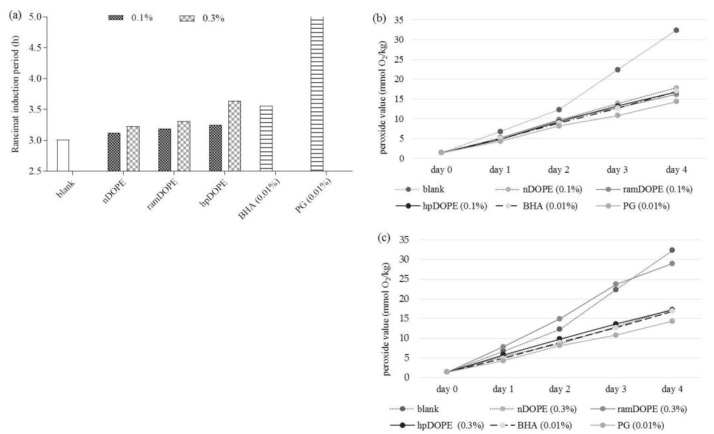
Antioxidant activity of DOPE in oil. (**a**): Rancimat induction period; (**b**): Peroxide value of safflower oil during storage with 0.1% DOPE; (**c**): Peroxide value of safflower oil during storage with 0.3% DOPE. nDOPE-native dry olive pomace extract; hpDOPE-extract prepared with hydroxypropyl-β-cyclodextrin; ramDOPE-extract prepared with randomly methylated β-cyclodextrin; BHA: Butylhydroxyanisol; PG: Propyl-gallate.

In this work, the ability of DOPEs to decrease lipid peroxidation of cold-pressed safflower oil was investigated by two accelerated oxidation approaches: the Rancimat method at 110 °C (Figure 1a) and the Shall oven test at 65 °C. (Figure 1b,c). In the Rancimat test, we tested the effect of the addition of DOPEs to oils at two concentration levels (0.1% and 0.3%) and the induction period was compared to the blank (pure safflower oil with no added antioxidants) and commonly used synthetic antioxidants (BHA and propyl-gallate (PG)).

The induction period for pure safflower oil was 3.01 h and was increased by the addition of all tested DOPEs at both concentration levels. Observed effects were concentration depended (addition of 0.3% DOPE showed higher protection effect in comparison to 0.1% DOPE) (Figure 1a). Among the tested DOPEs, nDOPE was the least potent. Cyclodextrin encapsulation significantly improved antioxidant efficiency DOPE in oil, from 2.3% (by addition of RAMEB) to 12.7% (by addition of HPβCD). The best antioxidant protection of safflower oil was were achieved by applying hpDOPE, especially at the concentration level of 0.3%, when it provided antioxidant efficiency comparable to 0.01% BHA. Recently, Mohammadi et al. [33] showed that significant antioxidant protection of soybean oil cannot be achieved by applying nano-encapsulated olive leaf extract (as determined by Rancimat test) but they used 3- to 10-fold lower sample concentrations in comparison to our investigation. PG provided the best oxidative stability during the Rancimat test and its efficiency at concentration level of 0.01% was approximately 60% higher in comparison to DOPEs at concentration levels of 0.1%. The main drawback of the Rancimat test in investigating the oxidative stability of plant oils is the fact that relatively high temperatures must be applied to obtain adequate sensitivity and selectivity of the method. However, at high temperatures, oxidation processes in oils are altered and can differ significantly from oxidation processes that occur under normal storage settings. For example, secondary oxidation reactions are favoured, and formation of peroxides becomes dependent on the oxygen concentration.

Therefore, the determination of the peroxide value measured under milder conditions (65 °C in Scholl Oven test) is probably a more reliable indicator of oxidation processes that occur under normal storage conditions. For that purpose, we monitored the increase of peroxide value of pure safflower oil (control) during the 4-day storage at 65 °C and compared it to results obtained for oil samples containing DOPEs or commonly used synthetic antioxidants (BHA and PG). Analysed DOPEs were applied at two concentrations levels 0.1% (Figure 1b) and 0.3% (Figure 1c). The presented results clearly show that addition of all DOPEs significantly reduced formation of lipid peroxides in comparison to the control. The efficiency of tested DOPEs (at both concentration levels) was comparable to or even higher than BHA (0.01%) as in the case of hpDOPE or ramDOPE (0.1%, 4th day of storage) (Figure 1b). Interestingly, applying higher concentration levels of all DOPEs (0.3%) except nDOPE increased peroxide formation in safflower oil indicating possible prooxidative effects. It was especially notable in the case of ramDOPE. Considering that this effect was only observed in samples encapsulated with CDs, it can be assumed that CDs (and especially RAMEB) might increase the content of lipid peroxides in oil. This observation is in discrepancy with the data obtained by the Rancimat method, where higher RAMEB level provided better antioxidant protection. The main difference of applied methods is that the Scholl oven test measures the peroxide value of the oil (primary reaction products) and the Rancimat method detects the content of secondary reaction products formed during the latter stages of lipid peroxidation. Therefore, the dose-dependent activity observed in the Rancimat test suggests that DOPE provides antioxidant protection of the oil primarily by inhibiting the formation of secondary reaction products. This is consistent with the recent findings of Lopez-Nicolas et al. [34], showing that cyclodextrins can be used as the potent inhibitors of secondary oxidation reactions in food (browning reactions).

#### 2.2.2. Antioxidant Activity of DOPE in Meat Model

Lipid oxidation is among the major factors contributing to quality deterioration of muscle food during storage, particularly due to production of aldehydes responsible for the development of rancid flavours and specific colour changes in meat [35]. Common antioxidants used by the food industry are highly efficient BHA and BHT. Recent discussions considering their safety and possible health implications of long term exposure have contributed to increasing consumer demand for more natural food-products, including antioxidants of natural origin. Among their main drawbacks is that they are more expensive than synthetic antioxidants since large amounts of plant materials are needed for their production and they need to be used in larger concentrations (since they are less effective than synthetic antioxidants). Therefore, novel approaches for extracting antioxidants from highly available food industry waste represents a sustainable and cost-effective concept.

**Figure 2:**
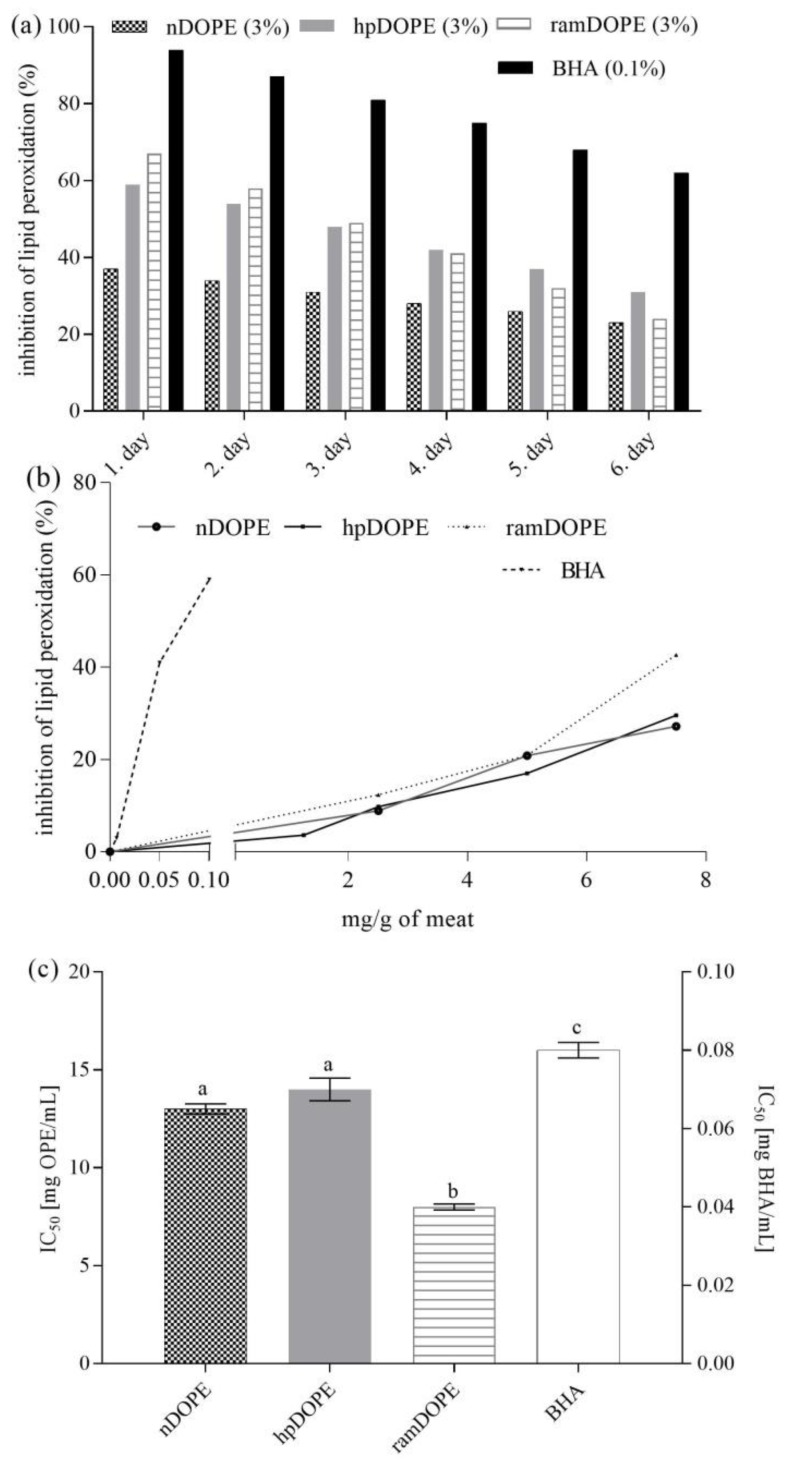
Oxidative stability of minced meat during storage at 4 °C and accelerate stability testing. (**a**): Oxidative stability during storage at 4 °C; (**b**): Inhibition of meat lipid peroxidation during accelerated testing in relation to concentration of antioxidant; (**c**): Antioxidant activity against meat lipid peroxidation during accelerated stability testing expressed as IC_50._ nDOPE-native dry olive pomace extract; hpDOPE-extract prepared with hydroxypropyl-β-cyclodextrin; ramDOPE-extract prepared with randomly methylated β-cyclodextrin; BHA: butylhydroxyanisol; IC_50_: Half maximal inhibitory concentration. Different letters above data bars indicate significant difference (*p* ≤ 0.05).

In this work, the efficiency of DOPEs as antioxidants in meats was assessed in minced meat by applying two approaches: stability testing during storage (Figure 2a) and accelerated stability testing and comparison to the activity of BHA (Figure 2b,c).

In both studies, the antioxidant potential of the tested antioxidants in meat was expresed as the inhibition of lipid peroxidation (relative to lipid peroxidation in meat sample with no added antioxidants) (Figure 2a,b). For stability testing during storage, meat samples containig no antioxidant (control), 3% DOPE, or 0.1% BHA were stored at 4 °C for 6 days and were analysed every day.

For accelerated stability testing, the impact of DOPE concentration (0–8 mg/g of meat) and BHA concentration (0–0.1 mg/g of meat) on inhibition of lipid peroxidation was monitored (Figure 2b). Half maximal inhibitory concentrations were calculated for each tested antioxidant and were used for the calculation of half minimal inhibitory concentration (IC_50_) (Figure 2c).

As can be seen from Figure 2a, in meat containing nDOPE, inhibition of lipid peroxidation ranged from 37% (day 1) to 23% (day 6) and was significantly lower in comparison to ramDOPE (67–24%) or hpDOPE (59–31%). Obtained results clearly point out that cyclodextrin encapsulation significantly increased the ability of DOPE to inhibit lipid peroxidation in meat during storage. However, the observed activity was low compared to 0.1% BHA which provided inhibition rates of 94–61%. Enhanced protective effects of cyclodextrin-encapsulated DOPE were also notable during accelerated stability testing. As expected, BHA was more potent than the tested DOPEs, providing 59% lipid peroxidation inhibition at the concnetration level of 0.1%. Among tested DOPE, ramDOPE was particularly potent, providing a 42.7% inhibition rate at the concentration level of 7.5 mg/g of meat (in comparison to 27.2% and 29.5% provided by nDOPE and hpDOPE, respectively) (Figure 2b). This resulted with low IC_50_ values obtained for ramDOPE (Figure 2c) showing that encapsulation with RAMEB is particularly effective in increasing antioxidant efficiency of DOPE while hpDOPE and nDOPE are significantly less potent. Discrepancies in the results obtained by two approaches (storage and accelerated stability testing) might be due to high temperatures used for accelerated stability testing (80 °C) probably affecting the stability of CD-polyphenol complexes or altering the kinetics and chemistry of lipid oxidation reactions naturally occurring in meat during storage.

Both approaches showed that extracts were effective in preventing lipid peroxidation in meat (compared to control); however, their efficiency was significantly lower than BHA. To achieve effects comparable to BHA (0.01%), DOPE had to be used at concentration levels of 1–3%. Those concentration levels are consistent with other published data; Tanabe et al. [36] proved the efficiency of 22 herbal extracts such as oregano, sage, thyme, etc. as antioxidants in meat applied in the concentration range 0.5–2.5% (used as freshly prepared liquid extracts) which is comparable with our data. Many other authors used medicinal plants and their freshly prepared extracts in similar concentration ranges for this purpose [37]. The advantage of our approach is that we used highly available raw material (food industry waste) for extract preparation, used green extraction techniques for extraction of antioxidants, and formulated a stable, dry extract that can be stored and is easy to manipulate. Therefore, applying DOPEs as natural alternatives to antioxidants used by the meat industry represents a cheaper, easier, and more sustainable approach.

#### 2.2.3. Antioxidant Activity of DOPE in the β-Carotene Emulsion Model System

**Figure 3:**
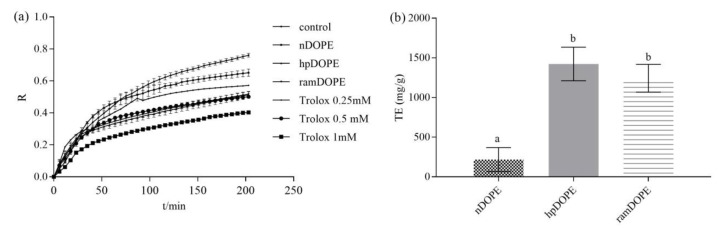
Antioxidant activity of DOPE in the β-carotene emulsion model system. (**a**): β-carotene absorbance inhibition ratio during 120 min incubation; (**b**): antioxidant activity expressed as IC_50._ nDOPE-native dry olive pomace extract; hpDOPE-extract prepared with hydroxypropyl-β-cyclodextrin; ramDOPE-extract prepared with randomly methylated β-cyclodextrin; BHA: butylhydroxyanisol; IC_50_: half maximal inhibitory concentration. Columns (in Figure 3b) marked with different letters belong to different statistical groups (*p* < 0.05)

A common practice in most antioxidant assays is to measure given responses of the sample and reference antioxidant at a single time. Measuring antioxidant activity at one predetermined time-point does not consider the kinetics of the process and unnecessarily simplifies the chemistry of the process, leading to incorrect results and misinterpretations. This is especially the case in investigations of complex natural extracts containing complex mixtures of anti- and pro-oxidants [37]. Therefore, the antioxidant activity of olive pomace extracts in the β-carotene-linoleate emulsion system was measured in a kinetic model by monitoring the absorption:inhibition ratio during 200 min of incubation (Figure 3).

Figure 3a presents β-carotene absorbance inhibition ratios during 120 min incubation in emulsion without added antioxidant (control), emulsions containing DOPEs, and emulsions containing different Trolox concentrations. Obtained data were used for development of Trolox calibration curve and obtained results were then expressed as mg/g of Trolox equivalents (Figure 2b).

Antioxidant activity of the analysed DOPEs ranged from 324.8 mg/g (nDOPE) up to 1272.4 mg/g and 1442.2 mg/g (hpDOPE and ramDOPE, respectively). This shows that the ability of analysed samples to inhibit the bleaching of β-carotene in the presence of hydroxyperoxide radicals formed from linoleic acid was particularly (approximately 4-fold) enhanced in cyclodextrin-encapsulated extracts. This is consistent with the results obtained in chemical tests for measuring antioxidant activity; however, both the antioxidative efficiency of DOPEs and the positive impact of cyclodextrins are much more pronounced in emulsion systems. For example, the ABTS^+^˙scavenging activity of analysed extracts was 12.86–17.07 mg/g TE, as opposed to 1509–1749 mg/g TE obtained in the β-carotene-linoleate emulsion system. The IC_50_ values of the analysed samples obtained by DPPH test ranged from 1.03–1.65 g/L, while in the β-carotene-linoleate emulsion system, a 50% increase of antioxidant protection was achieved with average DOPE concentrations around 0.125 g/L. The observed differences can be partially explained by the lower efficiency of Trolox, as a water-soluble form of vitamin E in oil-in-water emulsions, but also indicate the applicability of the tested DOPEs as antioxidants in such systems. Obtained results also point out significant limitations of the most commonly applied chemical approaches in assessing the applicability of natural extracts as potential antioxidants in foods. Namely, food models more closely reflect an oxidizing-food situation and can therefore provide more reliable data. Investigating efficiency in an oil/water emulsion system is very important since many food lipids exist as oil-in-water emulsions (e.g., milk products, dressings, beverages, ice cream, etc). They are especially susceptible to oxidation due to higher surface area that enables easier interaction of the oil with prooxidants present in the aqueous phase [38].

Our results are consistent with results obtained by Singh et al. [39] who showed that 0.1 g/L pomegranate peel and seed extracts provided a 20–40% increase of antioxidant protection in a β-carotene linoleate model system. Similar results were obtained for different Centaurea species extracts (1 g/L) by Zengin et al. [40].

### 2.3. Antioxidant Activity of DOPE in Biological Model Systems

#### 2.3.1. Inhibition of Plasmid DNA Strand Scission by DOPE

Free radicals produced by cellular metabolism or exogenous agents can react with biomolecules in cells, including DNA. The resulting oxidative damage to DNA is implicated in many biological processes such as mutagenesis, carcinogenesis, and aging [41]. Using plasmid DNA as a biological substrate presents an interesting step forward in developing non-cellular biological models for measuring antioxidant activity and has been used by several authors in evaluating biological activity of complex natural extracts [42,43].

The native conformation of plasmid DNA found in vivo is the supercoiled form, and is desired when isolating plasmid DNA. However, different external factors, such as free radicals, can change this native form into nicked relaxed circular plasmid, linear plasmid (when the DNA helix is cut in both strands at the same place), or circular single-stranded plasmid (due to disruption of hydrogen bonds). Different DNA forms show different mobility in agarose gel during electrophoresis: circular single strand is the fastest-migrating form, followed by the supercoiled form, linear, and nicked DNA, respectively.

In the DNA scission assay, 2,2′-azobis(2-amidinopropane) dihydrochloride radical (AAPH˙) was used to induce damage of the native supercoiled plasmid DNA and changes were monitored by agarose gel. In the control sample, DNA was present as supercoiled and to a significantly lesser extent, as nicked relaxed circular plasmid. Addition of AAPH˙ significantly decreased the content of the supercoiled and nicked forms and increased the content of linear form indicating significant oxidative damage (Figure S1).

**Figure 4:**
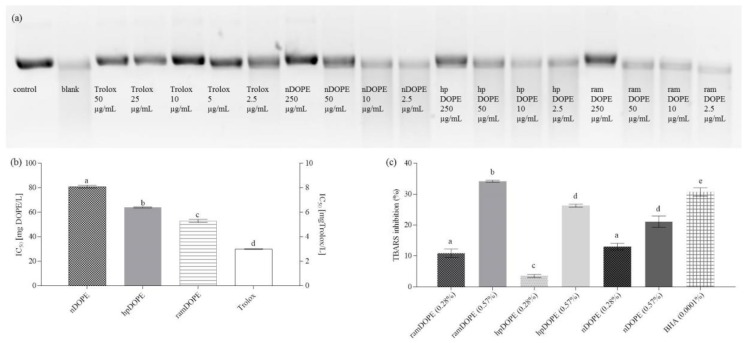
Inhibition of plasmid DNA strand scission and antioxidant activity of DOPE in liposome membrane model. (**a**): supercoiled (native) form of plasmid pBR322 DNA in electrophoresis gel after incubation with AAPH with or without the presence of antioxidants; (**b**): antioxidant activity against antioxidant activity against plasmid DNA strand scission expressed as IC50; (**c**): antioxidant activity of DOPE in liposome membrane model system. nDOPE-native dry olive pomace extract; hpDOPE-extract prepared with hydroxypropyl-β-cyclodextrin; ramDOPE-extract prepared with randomly methylated β-cyclodextrin; BHA: butylhydroxyanisol; IC_50_: half maximal inhibitory concentration; TBARS: thiobarbituric acid reactive substances. Different letters above data bars indicate significant difference (*p* ≤ 0.05).

As presented in Figure 4, addition of Trolox (2.5–50 µg/mL) and tested DOPEs (2.5–250 µg/mL) significantly reduced the damaging effect of AAPH in dose-dependent manner and enhanced the percentage of the plasmid DNA retained in native form. Applied concentrations of antioxidants were plotted against their respective supercoiled DNA retention percentages and used for the calculation of IC_50_ values (Figure 4b).

IC_50_ calculated for ramDOPE (53 mg/L) was similar to hpDOPE and significantly lower than nDOPE (64 and 81 mg/L, respectively), indicating higher protective potential. A possible mechanism that could explain the protective effect of tested extracts against DNA scission is the scavenging of reactive oxygen species, and the results obtained by antiradical activity testing chemical model systems (ORAC, ABTS, DPPH) support this theory (Table 2). Utilization of cyclodextrins in the formulation of dry extracts significantly improved the antioxidant activity of the final product, probably by increasing the content of hydroxytyrosol derivatives (HTD), major antioxidant species in DOPEs. Although the obtained IC_50_ values are high in comparison to the reference antioxidant (Trolox; IC_50_ = 3 mg/L), their effective concentrations are comparable to the activity of different plant extracts assessed by other authors [42,43], indicating potential for further investigations using cellular in vitro models and in vivo investigation.

#### 2.3.2. Antioxidant Activity of DOPE in the Liposome System

Liposomes are spherical phospholipid bilayers that mimic cellular structures and can be used as model membrane systems. They are widely used as xenobiotic delivery systems since they improve the availability of the active compound, target and slow its release, and show protective features [44]. This is why liposomes have potential applications in a wide range of fields, including pharmaceutical sciences, food technology, or agriculture. Additionally, they are used as model systems for investigation of membrane–drug interactions in terms of efficiency, toxicity, or pharmacokinetics [45]. As such, they provide a natural environment for studying the mechanisms of action and potency of biological antioxidants and can be used to predict their interaction with biological membranes and ability to protect them from oxidative degradation. They can also be used as models for measuring the ability of a particular antioxidant to inhibit lipid peroxidation and formation of thiobarbituric acid reactive substances (TBARS) during storage or heat treatment, and as such are a useful model for investigation in the food industry. Their relevance as cell-free liposome-based cellular models in investigating antioxidant activity has recently been emphasized by Balducci et al. [46].

In our work, liposomes were prepared by the sonication of a lipid dispersion and used for monitoring the ability of DOPE to inhibit lipid peroxidation induced by copper ions during storage at 37 °C (Figure 4c). Liposomes prepared without the addition of antioxidants were used as control and showed the highest rates of lipid peroxidation that was monitored by measuring thiobarbituric acid reactive substances (TBARS) production (data not shown). Production of TBARS was lower in liposomes containing antioxidants and their efficiency was expressed as the percentage of TBARS formation inhibition (relative to control). Tested DOPEs were applied at two concentration levels, 0.28% and 0.57%, respectively, and their efficiency was compared to 0.0001% BHA (Figure 4c).

At lower concentration levels, ramDOPE and nDOPE showed significantly higher inhibition rates (IR) against copper-induced lipid peroxidation in comparison hpDOPE (10.9 and 13.0 vs. 3.5%). At lower concentration levels nDOPE and ramDOPE showed the best antioxidant activity. At higher concentration levels, ramDOPE was still the most efficient among tested antioxidants and its IR was comparable to BHA that was used as control (34.2 vs. 30.7). The observed results can be explained primarily by tyrosol content of tested DOPEs (Table 2). Namely, among different phenolic compounds in olive pomace extract, hydroxytyrosol, tyrosol, and oleuropein are the most abundant (Table 1) and their content correlated well with TRC, ABTS antiradical activity, and ORAC (correlation coefficients were 0.8556, 0.8764, and 0.7838, respectively). Tyrosol is characterized with significantly higher logP in comparison to hydroxytyrosol and oleuropein (logP = 0.4 vs. −0.7 and −0.4, respectively). Tyrosol can therefore be most easily incorporated into hydrophobic interior of the liposome and inhibit lipid peroxidation. The content of tyrosol in ramDOPE (950.9 ± 7.61 mg/100 g) was significantly higher in comparison to nDOPE or hpDOPE (870.1 and 715.7 mg/100 g, respectively) resulting in significantly higher IR. However, hydrophilic antioxidants that remain in the outer solution and are not incorporated into liposome’s interior can also prevent its oxidation by the additive effect of their metal chelation ability. Namely, by chelating the copper ions in the solution, they inhibit the initiation of the oxidation process [47].

Our observations are consistent with the conclusions of Kong et al. [48] who used a liposome system to evaluate the antioxidant activity of various spice extracts. They showed that TBARS inhibition in oxidizing liposomes is correlated with total phenolic content and chelating activity of analyzed extracts, but not with DPPH**˙** scavenging activity, which is consistent with our data. Contribution of other characteristics of antioxidants in providing efficient protection in biological environment (in addition to their antiradical activity), such as self-assembly and their interaction with liposomes has been shown by Balducci et al. [46], showing that the chain-length of synthetized hydroxytyrosyl esters significantly affects their antioxidant activity in liposome model.

## 3. Materials and Methods

### 3.1. Chemicals and Reagents

Ethanol (96%) (Gram-mol, Zagreb, Croatia) was mixed with distilled water in order to obtain ethanol-water mixture used as extraction solvent. Acetonitrile (≥99.9%), methanol (≥99.9%), and formic acid used for the preparation of high performance liquid chromatography (HPLC) mobile phases and ultra-performance liquid chromatography-electrospray ionization-tandem mass spectrometry (UPLC-ESI-MS-MS) analysis were from Sigma-Aldrich (St. Louis, MO, USA). Reference standards of phenolic compounds 2-(4-hydroxyphenyl) ethanol (tyrosol > 99.5%), 3-hydoxytyrosol (≥98%), oleuropein (≥98%), 4-hydroxy-3-methoxybenzoic acid (vanillic acid), 4-hydroxy-3-methoxyphenylacetic acid (homovanillic acid), 3,4-dihydroxybenzoic acid, *p*-hydroxybenzoic acid were purchased from Sigma-Aldrich. Folin–Ciocalteu reagent, sodium carbonate, gallic acid, butyl-hydroxy anisole (BHA), phosphate buffer saline (PBS), 2,2-diphenyl-1-picrylhydrazyl (DPPH), 2,2′-azino-*bis*(3-ethylbenzothiazoline-6-sulphonic acid (ABTS), β-cyclodextrin (βCD) randomly-methylated β cyclodextrin (RAMEB), hydroxypropyl-β-cyclodextrin (HPβCD), γ cyclodextrin (γCD), 3′,6′-dihydroxyspiro[isobenzofuran-1[3*H*],9′[9*H*]-xanthen]-3-one (fluorescein), 2,2′-azobis-2-methyl-propanimidamide, dihydrochloride (AAPH), pBR322 Plasmid DNA from *Escherichia coli* RRI, malondialdehyde (MDA) tetrabutylammonium salt, 2-thiobarbituric acid, trichloroacetic acid, and human LDL (in PBS, pH 7.4, containing 0.01% of EDTA) were purchased from Sigma-Aldrich. GelRed^®^ dye for gel electrophoresis was obtained from Biotium (Fremont, CA, USA). Soy lecithin and other used chemicals were from Kemika (Zagreb, Croatia). Deionized water (18 mΩ) was obtained from Millipore-MiliQ water purification system (Merck, Kenilworth, NJ, USA).

### 3.2. Plant Material

**Scheme 1:**
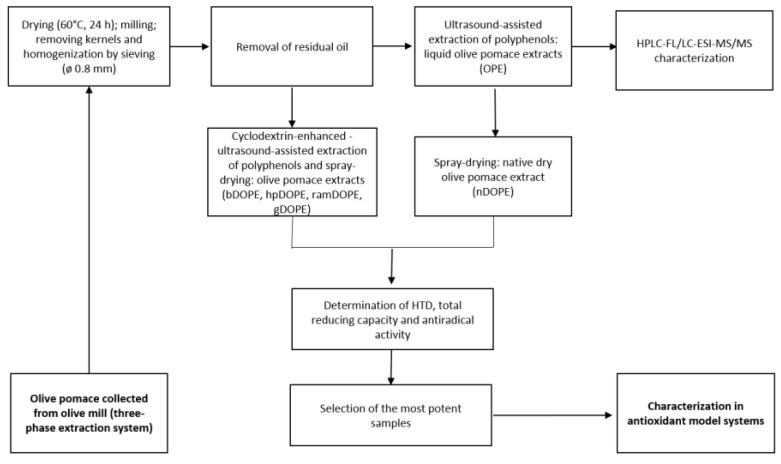
Flowchart of the experimental design (preparation of extracts and analyses). HTD-hydroxytyrosol derivatives; nDOPE: native dry olive pomace extract; hpDOPE: extract prepared with hydroxypropyl-βcyclodextrin; ramDOPE-extract prepared with randomly methylated βcyclodextrin; bDOPE: extract prepared with βcyclodextrin; gDOPE: extract prepared with γ-cyclodextrin.

Olive pomace was collected from several three-phase olive-mill plants during winter 2016/2017. As described previously [15], plant material pretreatment included drying, milling, removing kernels homogenization, and de-fatting in a Soxhlet apparatus in order to obtain powder suitable for the extraction of polyphenols (Scheme 1).

### 3.3. Preparation of Olive Pomace Extracts

Extraction of polyphenols was conducted according to previously optimized procedure [14]. Briefly, powdered olive pomace was extracted using 60% ethanol at 1:40 solvent to solid ratio using ultrasound-assisted extraction (Q Sonica Sonicators, Newtown, CT, USA). For maximal yields, 20 min pulsed extraction was applied, using 12 mm titanium probe at 100% output intensity. For CD-enhanced extraction, cyclodextrins were added to extraction solvent at concentration levels 8 g/L (βCD) or 16 g/L (HPβCD, RAMEB, γCD), as previously suggested [14]. Obtained liquid extracts were spray-dried using Mini Spray Dryer B-290 (Büchi, Flavil, Switzerland) in combination with either βCD, HPβCD, RAMEB, or γCD encapsulation or without the addition of cyclodextrin under previously optimized conditions (*unpublished data*). Different types of DOPEs were obtained: nDOPE, hpDOPE, ramDOPE, dry extract encapsulated with βCD (bDOPE), and dry extract encapsulated with γCD (gDOPE).

### 3.4. Chromatographic Analysis of Olive Pomace Extract

#### 3.4.1. HPLC Analysis of Hydroxytyrosol Derivatives

Identification and determination of the main hydroxytyrosol derivatives (tyrosol, hydroxytyrosol, and oleuropein) was conducted by Waters 2695 HPLC system (Waters Milford, MA, USA) coupled with a 2475 multi fluorescence detector (FLD), according to method of Tsarbopoulos et al. [49]. Samples were dissolved in deionized water and filtered through 0.45 µm syringe filters (polyethersulfone membrane, Macherey-Nagel, Düren, Germany). Chromatographic separation was achieved on a C18 reversed phase column (250 × 4.6mm, 5 µm) (Agilent Zorbax Eclipse plus, Agilent Technologies, Santa Clara, CA, USA). The gradient elution program was applied for satisfactory separation of peaks (Table S1). The mobile phase used was 0.05 mol/L ammonium acetate buffer (adjusted to pH 5.0 with glacial acetic acid) versus acetonitrile. The flow rate was 1 mL/min and the injection volume was 20 μL. The FLD was set at the excitation wavelength of 280 nm, and emission wavelength of 635 nm. The column temperature was maintained at 25 °C. The identification of tyrosol, hydroxytyrosol, and oleuropein was carried out by the comparison of the retention times of pure standards. The quantification of the analytes was performed by external standard calibration. Standard stock solutions were prepared by dissolving reference compounds in methanol. Aliquots of these solutions were further diluted with to obtain calibration standards at concentrations between 1–81 mg/L (five concentration points: 1, 3, 9, 27, and 81 ppm). Identification of the eluting peaks was performed by comparing their retention times with those of the standards.

#### 3.4.2. UPLC–ESI-MSMS Analysis of Phenolic Acids

The phenolic compounds were analyzed by Agilent series 1290 RRLC instrument (Agilent Technologies, Santa Clara, CA, USA) coupled to an Agilent triple quadrupole mass spectrometer (6430) with ESI ion source (UPLC-MS/MS). Analysis was conducted according to the method of López de las Hazas et al. [50] with some modifications: instead of 0.2% (*v*/*v*) acetic acid, 0.1% (*v*/*v*) formic acid was used and flow rate was reduced to 0.2 mL/min. Ionization was done by electrospray (ESI) in the negative mode, and the data were collected in the dynamic MRM mode. The MS/MS parameters were as follows: capillary voltage, 4000 V in positive and 3500 V in negative acquisitions; nebulizer pressure 40 psi; drying gas temperature 300 °C; gas flow rate 11 L/min. Nitrogen (>99% purity) was used as the nebulizing and collision gas, respectively. Precursor and product ions were identified and optimized using Mass Hunter Optimizer (Agilent Technologies, Santa Clara, CA, USA). Different phenolic compounds were characterized according to their mass spectra and according to retention times that were compared with commercial standards. All standards were qualified and quantified in dynamic multiple reaction monitoring (MRM) mode, using the optimized specific parameters: precursor ion, product ion, fragmentor voltage, collision energy, and ionization mode (Table S2). Quantification was performed using the external standard method. Standard stock solutions were prepared by dissolving reference compounds in methanol and were adequately diluted with deionized water to obtain calibration standards at concentrations between 0.1–10 mg/L. Samples were sonicated for 30 min with methanol in the ultrasonic bath at constant frequency of 37 kHz and at temperature of 50  ±  5 °C (Model Elma, Elmasonic S 40 (H), Singen, Germany), centrifuged at 3000 rpm for 15 min, and filtered through 0.45 μm filter.

### 3.5. Determination of Antioxidant Activity in Chemical Model Systems

#### 3.5.1. Determination of Total Reducing Power

The quantification of the total phenolic content was carried out by Folin–Ciocalteu method according to Singleton and Rossi [51] with some modifications. The reaction mixture consisted of 200 µL of adequately diluted extract or extraction medium (blank), 1.35 mL of deionized water and 150 µL of Folin–Ciocalteu reagent. Reaction mixture was vortexed. After 5 min, 1.5 mL of Na_2_CO_3_ (6%, *w*/*v*) was added and the mixture was shaken in water bath (70 rpm) for 30 min, at 50 °C. Absorbance was measured at 725 nm, after cooling to room temperature. Calibration curve was prepared with gallic acid and total reducing capacity was expressed as mg of gallic acid equivalents (mg/g GAE).

#### 3.5.2. DPPH Radical-Scavenging Assay

The radical-scavenging activity against DPPH**˙** was determined according to method of Kirigaya et al. [52] and Shimada et al. [53]. DPPH**˙** radical scavenging activity is method based on the reduction of DPPH**˙** in methanol solution in the presence of a hydrogen-donating antioxidant that results with discoloration of purple solution that can be measured at 528 nm [54]. The reaction mixture consisted of 600 µL of adequately dissolved extract in methanol, or 600 µL of pure methanol for estimating initial absorbance (*A_0min_*), 1.4 mL of methanol, and 1.5 mL of DPPH solution. It was incubated for 30 min and the absorbance was measured at 528 nm (*A_30min_*). Each sample was analyzed at 4–5 concentration levels, so that the decrease of the absorbance varied between 10% and 90%. Absorbance decrease (%) was plotted against the concentration of particular antioxidant and obtained equations were describing observed linear dependence were used for the calculation of IC_50_, which represents a concentration of extract that caused the 50% absorbance decrease.

#### 3.5.3. Scavenging Activity against the ABTS Radical (TEAC Test)

Scavenging activity of olive pomace extracts against the ABTS^+˙^ was determined by a colorimetric assay described by Re et al. [55]. ABTS^+·^ solution was prepared by 7 mmol/L aqueous ABTS solution reacting with 2.45 mmol/L potassium persulfate solution in the dark at 4 °C for 12 h. After reaction, ABTS^+^ solution was diluted with distilled water to give an absorbance of 0.70 ± 0.02 at 732 nm. The reaction mixture consisted of 300 µL of adequately diluted extract or deionized water (*A_0min_*) and 2.5 mL ABTS^+·^ solution. Absorbance of the samples was measured at 732 nm, after 3 min of reaction (*A_3min_*). The percentage of quenching the absorbance was calculated according to the Equation (Eq1) [1]:

(1) $$\;\Delta A=\frac{A_{0\min}-A_{3\min}}{A_{0\min}}\times 100\;$$

The calibration curve was generated by plotting different Trolox concentrations against respective absorbance quenching percentages. Antiradical efficiency was expressed as mg of Trolox equivalents (mg/g TE).

#### 3.5.4. Oxygen Radical Absorbance Capacity (ORAC) 

Oxygen radical absorbance capacity (ORAC) measures free radical oxidation of a fluorescent probe through the change in its fluorescence intensity. It was determined using fluorescein as the fluorescent probe [56]. The assay was performed in a 96-well microplate and was assessed with a Victor X3 Multilabel Plate Reader (Perkin Elmer, Waltham, MA, USA). Briefly, 150 mL of 5 µM fluorescein was added to each well of a black microplate and mixed with 25 µL of phosphate buffer (75 mM, pH 7.0; blank), Trolox standard (6.25–100 µmol/L) or adequately diluted sample. The reaction mixture was incubated at 37 °C for 10 min and the oxidation reaction was initiated by addition of 25 µL of AAPH (150 mM in phosphate buffer). The fluorescence of the reaction mixtures (excitation 485 nm, emission 530 nm) was plotted against time (60 min).

The area under the curve (AUC) was calculated for blank, samples, and standard (Trolox). For each sample and each Trolox concentration, netAUC was calculated by subtracting AUC_blank_ from AUC_sample/Trolox_. A linear standard curve was generated by plotting Trolox concentrations against respective netAUC values. The obtained standard curve was used to express the antioxidant activity of the samples as mg of Trolox equivalents per gram (mg/g TE).

### 3.6. Antioxidant Activity in Food Model Systems

#### 3.6.1. Antioxidant Activity in Oil

Oil Thermal Oxidative Stability Index (Rancimat Test)

Oxidative stability of oils containing different amounts of DOPEs was investigated by determining the induction period (IP) on a Rancimat 743 apparatus (Metrohm, Herisau, Switzerland) at 110 °C and an air flow of 20 L/h [57]. The Rancimat test measures the conductivity of deionized water caused by dry air that bubbled through a heated sample and automatically determines the time needed to produce maximal conductivity [58]. 3 g of oil containing the tested samples or synthetic antioxidants were weighed into reaction vessels and analyzed simultaneously. The IP was determined for all sample oils. The IP has been analyzed in triplicate.

Schaal Oven Stability Test 

Oil samples were submitted to an accelerated storage test [59] which was conducted in a forced-draft oven at 63 °C for 4 days. Peroxide value (PV), as the marker of oxidative stability of safflower oil was determined in fresh oil, and after 1, 2, 3, and 4 days. All determinations were carried out in duplicate.

#### 3.6.2. Antioxidant Activity in Meat Model System

Antioxidant activity in meat model system was determined according to the modified procedure described by Fasseas et al. [35]. In this method lipid peroxidation is determined by the reaction of MDA with thiobarbituric acid (TBA) to form a colorimetric (532 nm) product proportional to the MDA present. MDA tetrabutylammonium salt was used for the preparation of standard curve. Minced meat was homogenized, and visible fat was removed. Meat samples, with or without (control) the addition of DOPE/BHA were subjected to either thermal processing or storage at 4 °C. For determination of lipid peroxidation products, samples were mixed with 1 mL of water, 1.5 mL of acetic acid (20%, *v*/*v*), and 1.5 mL of 0.8% (*w*/*w*) TBA in 1,1% (*w*/*w*) sodium-dodecyl-sulphate (pH of reaction mixture was 2). Reaction mixture was vortexed for 30 s and incubated in water bath for 60 min at 100 °C. After cooling, 5 mL of butan-1-ol was added to reaction mixture and vortexed for 60 s. Reaction mixtures were centrifuged at 4000 rpm for 3 min. The absorbance of the butanol layer was determined at 532 nm and butan-1-ol was used as blank.

For accelerated stability testing, meat samples without added antioxidant, containing different concentrations of DOPEs or containing BHA, were shaken in water bath (100 rpm) for 120 min, at 85 °C. Inhibition of MDA products formation (%) was plotted against the concentration of added antioxidant DOPE (0.25–0.75%) or BHA (0.005–0.1%) and results were expressed as the IC_50_.

Stability during storage was assessed by daily sampling of meat stored at 4 °C in a dark over 6 consecutive days. 3 mg of DOPE or 0.1 mg of BHA was added to 100 mg of meat, while meat stored without the addition of antioxidants was used as control. The results were expressed as the percentage of the inhibition of formation of lipid peroxide radicals (quantified as MDA) in relation to control.

#### 3.6.3. Antioxidant Activity in β-Carotene Emulsion Model System

The antioxidant activity of the extracts in the β-carotene emulsion model system was determined by the improved method of Prieto et al. [37] in a microtiter plate, using a kinetic approach. For the preparation of the β-carotene–linoleic acid emulsion β-carotene (4 mg), linoleic acid (0.5 mL), and Tween-40 (4 g) were dispersed in 20 mL of chloroform and shaken to obtain a suspension. The chloroform was evaporated at a temperature lower than 50 °C in a short period of time to avoid the beginning of the lipid oxidation process as much as possible. 1 mL of the oily residue was mixed with 30 mL of 100 mmol/L Briton buffer (pH 6.5, preheated at 45 °C) to prepare emulsion. The subsequent procedure was performed in a 96-well microplate with flat-bottom wells by combining 50 μL of DOPE (0.125 g/L), buffer (blank), or Trolox (standard; 0.125 mmol/L) and 250 µL of emulsion. The reader device was programmed to 45 °C with agitation (660 s/min and 2 mm amplitude). The absorption of reaction mixture was monitored every minute for 200 min at 470 nm using the Victor X3 Multilabel Plate Reader (Perkin Elmer, Waltham, MA, USA). Absorbance inhibition ratios (R) were calculated according to the Equation (Eq2) [2]:

(2) $$\;R=1-\frac{A_{0}}{A_{t}}\;$$

where *A_0_* is maximal absorbance of reaction recorded at time 0 and *A_t_* is absorbance recorded at time *t* (1–200 min). *R* was plotted against time (Figure 3a) and obtained values were used for calculation of area under the curve (AUC) for each analysed sample/Trolox solution. Linear regression curve was generated by plotting AUC with respective Trolox concentrations (0.125–1 mM) and obtained regression equation was used to calculate and express antioxidant activity of analyzed samples as mg/g of Trolox equivalents (TE).

### 3.7. Antioxidant Activity in Biological Model Systems

#### 3.7.1. Inhibition of Plasmid DNA Strand Scission

Inhibition of DNA scission in plasmid pBR322 was measured according to the procedure modified from Kitts and Wijewickreme [60]. Briefly, 4 µL of pBR322 plasmid DNA (50 µg/L) was mixed with 2 µL of extract solution (different dilutions; sample), PBS (10 mmol/L; pH = 7.4; blank), or Trolox (different concentrations; control); 2 µL of AAPH˙ (50 mmol/L) or PBS (control). The final volume of the reaction mixture was brought to 10 µL with PBS. Following incubation (37 °C, 120 min, 350 rpm), 4 µL of loading dye (2.5 g/L of bromophenol blue; 40 g/L saccharose; 10 mL/L GelRed dye) was added to reaction mixture and loaded onto 7 g/L of agarose gel. After electrophoresis DNA bands were visualized and quantified with Image Quant LAS 500 (GE Healthcare Life Sciences, Amersham, UK). Antioxidant activity was assessed based on the percentage of DNA remaining in its native supercoiled form after incubation with AAPH˙ radical. Based on regression equation that has been calculated by plotting the sample concentration in the reaction mixture (10–250 mg/L) against the percentage of intact DNA, IC_50_ has been established for each analyzed sample.

#### 3.7.2. Antioxidant Activity in Liposome Model System

Antioxidant activity in liposome model system was determined according to the modified procedure described by Liu et al. [61]. Briefly, 2.4 g of soy lecithin was suspended in 300 mL of deionized water (8 mg/mL) by stirring and sonicating for 15 min. 1 mL of antioxidant ethanol solutions (BHA or DOPEs) or ethanol (control sample) was added to 50 mL of liposome and the mixture was sonicated for 5 min. 20 µL of cupric acetate (30 mmol/L) was added to the solution and reaction mixture was mixed thoroughly. The antioxidant activity of DOPE was measured at two concentration levels: 0.28% and 0.57%.

Liposome solutions were incubated in water bath for 60 min, at 37 °C. After cooling to room temperature, 1 mL of liposome solution was mixed with 1.5 mL of acetic acid (20%, *v*/*v*) and 1.5 mL of 0.8% (*w*/*w*) TBA in 1.1% (*w*/*w*) sodium-dodecyl-sulphate (pH of reaction mixture was 2). Reaction mixture was vortexed for 30 s and incubated in water bath for 60 min at 100 °C. After cooling, 5 mL of butan-1-ol were added and mixed. Samples were then centrifuged at 4000 rpm for 3 min. The absorbance of the upper layer was determined at 540 nm and butan-1-ol was used as a blank. Antioxidant activity was expressed as the percentage of inhibition of TBARS formation in relation to control sample).

### 3.8. Statistical Analysis

All experiments were performed in triplicate. The results were expressed as mean ± SD. Statistical comparisons among the analyzed samples were made using one-way analysis of variance (ANOVA) followed by Dunnett’s post-hoc test. *p* < 0.05 was considered statistically significant. Statistical analyses were performed using the demo version of GraphPad Prism (GraphPad Software, La Jolla, CA, USA, www.graphpad.com).

## 4. Conclusions

This study explored the possibilities of utilizing olive pomace extracts as antioxidants in different models and investigated the effect of cyclodextrin encapsulation on the activity of the final formulation. Cyclodextrin encapsulation enhanced the antioxidant activity of olive pomace extracts by significantly increasing their polyphenolic content, especially in the case of hydroxytyrosol derivatives (hydroxytyrosol, tyrosol, and oleuropein). Other proposed mechanisms of positive cyclodextrin effects on antioxidant activity are the modification of physical characteristics and increasing the stability of active substances under oxidative conditions. The tested cyclodextrins significantly differed regarding their ability to improve the functionality of olive pomace extracts; encapsulation with HPβCD and RAMEB provided comparable and the most significant benefits. Applying food and biological models for testing antioxidant activity revealed more significant differences in antioxidant activity between native and cyclodextrin-encapsulated extracts in comparison to chemical models, suggesting their applicability for testing the activity of substance/extract in natural environments and their interaction with biological substrates. hpDOPE and ramDOPE were particularly potent in oil-in-water emulsion systems, showing higher antioxidant activity than Trolox. Their antioxidant activity in the oil and meat models was comparable to that of BHA when applied at concentration levels of 0.1% and 2–3%, respectively. ramDOPE showed significantly higher antioxidant activity in food models (in comparison to hpDOPE). In biological models, they showed the ability to inhibit lipid peroxidation by direct radical scavenging capacity and metal-chelating properties and successfully protected DNA from AAPH˙ induced scission. The obtained results contribute significantly to the current knowledge on the antioxidant activity of olive pomace extracts, their potential application as nutraceuticals, and significant benefits that can be provided by the simple process of cyclodextrin encapsulation.

## Figures and Tables

**Table 1:** Contents of main phenolic compounds in DOPE.

Hydroxytyrosol Derivatives (mg/100 g)		Phenolic Acids (mg/100 g)	
Hydoxytyrosol	51.26 ± 0.40 ^A^	3,4-dihydroxybenzoic acid	3.72 ± 0.47 ^A^
Tyrosol	88.67 ± 1.17 ^B^	vanillic acid	2.16 ± 0.13 ^B^
Oleuropein	16.29 ± 0.39 ^C^	homovanillic acid	1.25 ± 0.12 ^C^
		*p*-hydroxybenzoic acid	0.33 ± 0.058 ^D^

All tests were performed in triplicate (*n* = 3); results are means ± SD of parallel tests. For each group of compounds, means in a column followed by different letters (from ^A^ to ^D^) are significantly different at *p* < 0.05.

**Table 2:** Impact of cyclodextrins on the content of hydroxytyrosol derivatives and chemical antioxidant activity of olive pomace extracts.

	nDOPE	hpDOPE	ramDOPE	bDOPE	gDOPE
Hydroxytyrosol (mg/kg)	513.1 ± 4.77 ^A^	716.8 ± 16.97 ^B^	663.6 ± 39.03 ^B^	497.4 ± 5.67 ^A^	527.1 ± 12.87 ^A^
Tyrosol (mg/kg)	870.1 ± 35.07 ^A^	715.7 ± 19.92 ^B^	950.9 ± 7.61 ^C^	804.9 ± 0.33 ^AC^	777.7 ± 7.65 ^BC^
Oleuropein (mg/kg)	166.0 ± 7.45 ^A^	249.4 ± 5.93 ^B^	157.6 ± 12.96 ^A^	171.4 ± 1.00 ^A^	226.8 ± 10.19 ^B^
HTD (mg/100 g)	1549.2 ± 22.9 ^A^	1682 ± 8.9 ^B^	1772.1 ± 44.4 ^B^	1473 ± 4.3 ^A^	1531 ± 30.7 ^A^
TRC (mg/g GAE)	39.24 ± 0.042 ^A^	50.97 ± 0.516 ^B^	53.16 ± 0.164 ^C^	44.21 ± 0.878 ^D^	42.71 ± 0.45 ^D^
IC_50_ against DPPH (g/L)	1.65 ± 0.129 ^A^	1.039 ± 0.129 ^B^	1.032 ± 0.094 ^B^	1.175 ± 0.007 ^B^	1.429 ± 0.083 ^A^
TEAC (mg/g TE)	12.86 ± 0.419 ^A^	15.44 ± 0.133 ^B^	17.07 ± 0.51 ^C^	14.00 ± 0.33 ^A^	12.71 ± 0.24 ^A^
ORAC (mg/kg TE)	152.8 ± 5.06 ^AB^	166.5 ± 7.51 ^A^	165.8 ± 6.34 ^A^	142.7 ± 10.21 ^B^	162.8 ± 10.24 ^A^

Results are expressed as means ± standard deviations of four parallel investigations (*n* = 4). Different letters in the same row indicate significant difference at *p* < 0.05. HTD-hydroxytyrosol derivatives (sum of hydroxytyrosol, tyrosol, and oleuropein); GAE-gallic acid equivalents; TRC: total reducing capacity; DPPH-2,2-diphenyl-1-picrylhydrazyl; TE-Trolox equivalents; TEAC: Trolox equivalent antioxidant activity; ORAC: oxygen radical activity test; nDOPE-native dry olive pomace extract; hpDOPE-extract prepared with hydroxypropyl-β-cyclodextrin; ramDOPE-extract prepared with randomly methylated β-cyclodextrin; bDOPE: extract prepared with β-cyclodextrin; gDOPE: extract prepared with γ-cyclodextrin.

## Supplementary Materials

The following are available online. Figure S1: Plasmid pBR322 DNA forms visible after electrophoresis in agarosis gel in the presence or absence of free radicals (AAPH) and/or antioxidants (Trolox), Table S1: HPLC elution program (gradient of mobile phase and flow rate). A mobile phase is sodium acetate buffer (pH 5.0), and B is acetonitrile, Table S2: ESI-MS/MS parameters for studied phenolic compounds.
