# Supplementary material for: Valorization of Olive Pomace-Based Nutraceuticals as Antioxidants in Chemical, Food, and Biological Models
Source: Molecules. 2018 Aug 18;23(8):2070. doi: 10.3390/molecules23082070 (PMC6222651; doi:10.3390/molecules23082070)
Supplement: Supplementary file 1 [file molecules-23-02070-s001.pdf]

Table S1. HPLC elution program (gradient of mobile phase and flow rate). A mobile phase is sodium acetate buffer (pH 5.0), and B is acetonitrile.

t [min]	Flow rate [mL/min]	A [%]	B [%]
0	1	90	10
4	1	80	20
8	1	40	60
11	1	30	70
15	1	90	10
20	1	90	10

A: sodium acetate buffer (pH 5.0); B: acetonitrile.

Table S2. ESI-MS/MS parameters for studied phenolic compounds.

Compound	Rt (min)	Precursor ion (m/z)	Product ion (m/z)	Cone voltage (V)	Collision energy (V)	Ionization mode
3,4-dihydroxybenzoic acid	7.189	153	109	70	10	-
p-hydroxybenzoic acid	9.665	137	93	90	5	-
Homovanillic acid	10.366	150.9	91/119	100	15	+
Vanillic acid	10.741	168.9	93	100	5	+

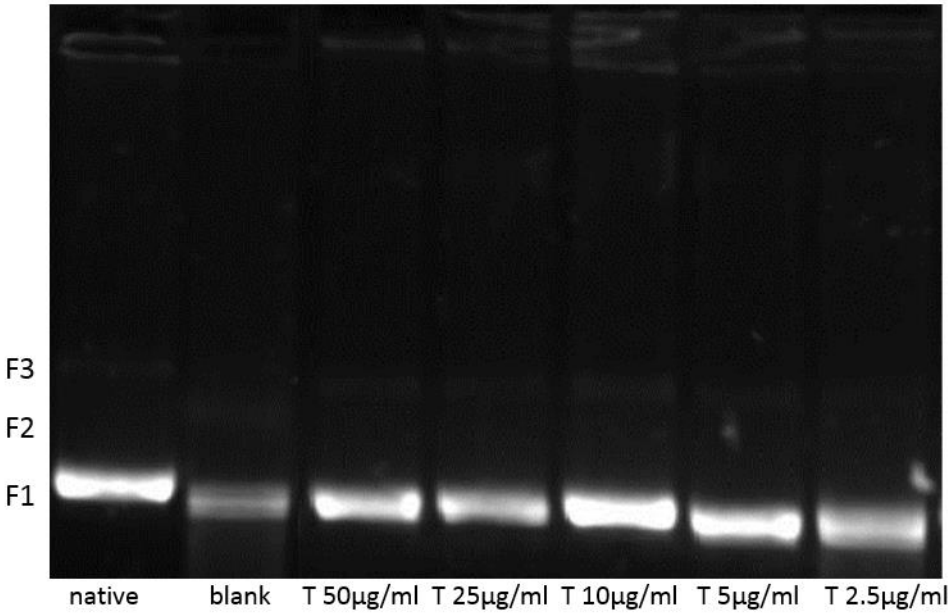

Figure S1. Plasmid pBR322 DNA forms visible after electrophoresis in agarosis gel in the presence or absence of free radicals (AAPH) and/or antioxidants (Trolox). native sample (DNA in phosphate buffer); blank (DNA+AAPH; no antioxidants); T (DNA+AAPH+Trolox); F1: native, supercoiled form; F2: linear plasmid; F3: nicked relaxed circular plasmid.
